# Elevated Monocytic Interleukin-8 Expression under Intermittent Hypoxia Condition and in Obstructive Sleep Apnea Patients

**DOI:** 10.3390/ijms222111396

**Published:** 2021-10-22

**Authors:** Li-Pang Chuang, Huang-Pin Wu, Li-Ang Lee, Li-Chung Chiu, Shih-Wei Lin, Han-Chung Hu, Kuo-Chin Kao, Ning-Hung Chen, Jung-Wei Tsai, Jong-Hwei Su Pang

**Affiliations:** 1 Sleep Center, Department of Pulmonary and Critical Care Medicine, Chang Gung Memorial Hospital, Linkou, Taoyuan 333423, Taiwan; lpchuang@gmail.com (L.-P.C.); pomd54@cgmh.org.tw (L.-C.C.); ec108146@cgmh.org.tw (S.-W.L.); h3226@cgmh.org.tw (H.-C.H.); kck0502@cgmh.org.tw (K.-C.K.); ninghung@yahoo.com.tw (N.-H.C.); jw1980.tsai@msa.hinet.net (J.-W.T.); 2 Department of Respiratory Therapy, Chang Gung University, Taoyuan 333323, Taiwan; 3 School of Medicine, Chang Gung University, Taoyuan 333323, Taiwan; 5738@cgmh.org.tw; 4 Department of Pulmonary and Critical Care Medicine, Chang Gung Memorial Hospital, Keelung 204201, Taiwan; whanpyng@cgmh.org.tw; 5 Department of Otorhinolaryngology-Head and Neck Surgery, Chang Gung Memorial Hospital, Linkou, Taoyuan 333423, Taiwan; 6 Graduate Institute of Clinical Medical Sciences, College of Medicine, Chang Gung University, Taoyuan 333323, Taiwan; 7 Department of Physical Medicine and Rehabilitation, Chang Gung Memorial Hospital, Taoyuan 333423, Taiwan

**Keywords:** chemotaxis, interleukin 8, intermittent hypoxia, monocyte, obstructive sleep apnea

## Abstract

Obstructive sleep apnea (OSA) is a disease with great cardiovascular risk. Interleukin-8 (IL-8), an important chemokine for monocyte chemotactic migration, was studied under intermittent hypoxia condition and in OSA patients. Monocytic THP-1 cells were used to investigate the effect of intermittent hypoxia on the regulation of IL-8 by an intermittent hypoxic culture system. The secreted protein and mRNA levels were studied by means of enzyme-linked immunosorbent assay and RT/real-time PCR. The chemotactic migration of monocytes toward a conditioned medium containing IL-8 was performed by means of the transwell filter migration assay. Peripheral venous blood was collected from 31 adult OSA patients and RNA was extracted from the monocytes for the analysis of IL-8 expression. The result revealed that intermittent hypoxia enhanced the monocytic THP-1 cells to actively express IL-8 at both the secreted protein and mRNA levels, which subsequently increased the migration ability of monocytes toward IL-8. The ERK, PI3K and PKC pathways were demonstrated to contribute to the activation of IL-8 expression by intermittent hypoxia. In addition, increased monocytic IL-8 expression was found in OSA patients, with disease severity dependence and diurnal changes. This study concluded the monocytic IL-8 gene expression can be activated by intermittent hypoxia and increased in OSA patients.

## 1. Introduction

Obstructive sleep apnea (OSA) is a highly prevalent clinical disease affecting more than 10% of the adult population [1]. It is characterized by repetitive episodes of partial or total upper airway obstruction during sleep, resulting in subsequent sleep fragmentation and intermittent hypoxia [2]. Accumulating studies reveal that OSA is an independent risk factor for hypertension and consequent cardiovascular morbidities, such as myocardial infarction, heart failure, nocturnal dysrhythmias and pulmonary hypertension [3]. Currently, OSA can be treated by means of positive airway pressure therapy, oxygen therapy and pharmacological therapy [4]. Surgical intervention should be considered in patients who are noncompliant with the above treatments or in whom they fail [5]. Barbed suture pharyngoplasty, one of the updated surgical technique, has been shown to be effective in OSA patients in controlling the autonomic function of the heart, demonstrating a decrease in sympathetic activity after surgery significantly associated with surgical success and a decrease in AHI (*p* = 0.033 and *p* = 0.001, respectively) [6,7].

Coronary heart disease, one of those sequelae of OSA, results from the accumulation of atheromatous plaques in the walls of coronary arteries [8]. Some studies have shown high prevalence of coronary heart disease among patients with OSA and vice versa [9,10]. Current evidence demonstrates the activation of inflammatory pathways in circulating monocytes by intermittent hypoxia, the important characteristic of OSA, which is a critical step that induces injury of the endothelium [11,12]. The adhesion and transmigration of monocytes through the vascular endothelial layer are initiated by attraction by chemokines, resulting in the development of atherosclerosis [13].

Interleukin-8 (IL-8) is a well-known chemoattractant response to the chemotaxis of circulating leukocytes [14]. IL-8 can mediate the accumulation of macrophages in atherosclerotic lesions [15]. One study reported that IL-8 is involved in the initial contact of monocytes with the endothelium and another study reported that IL-8 is involved in the adhesion of monocytes to the endothelium [16,17]. Although it has been known that macrophages can produce IL-8, macrophages in the atherosclerotic lesions of mice robustly express its receptor, CXCR2 [18]. The IL-8/CXCR2 pathway plays an important role in the trafficking and accumulation of macrophages in the vessel wall, which was proven by the reduced macrophage content of the atherosclerotic plaque in mice lacking CXCR2 compared with normal CXCR2 mice [18].

Although some literature sources have shown the increased circulating IL-8 levels in OSA patients [19,20,21], there are still puzzles in understanding the possible source and mechanism leading to this phenomenon. Furthermore, no currently published literature has mentioned whether “intermittent hypoxia” can activate monocytes to express more IL-8, which facilitates the subsequent formation of atherosclerosis. Therefore, we conducted this study to evaluate the effect of intermittent hypoxia on monocytic IL-8 expression and the related signal pathways involved in this regulation. We also examined the IL-8 expression in the monocytes isolated from OSA patients to investigate the diurnal changes of the sleep apnea effect. We hypothesized that intermittent hypoxia could activate monocytes to enhance IL-8 production which contributes to the increased plasma IL-8 level in OSA patients.

## 2. Results

### 2.1. Intermittent Hypoxia Promoted the IL-8 Protein Production and mRNA Expression in the Monocytic THP-1 Cells

**Figure 1:**
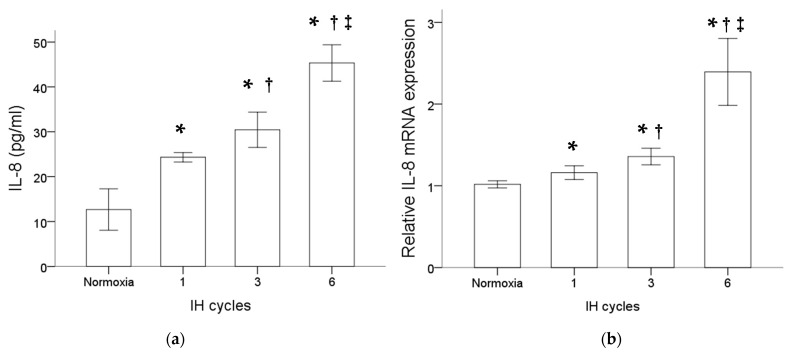
Intermittent hypoxia enhanced IL-8 protein secretion and gene expression in monocytes. THP-1 cells were treated with normoxia or intermittent hypoxia for one, three and six cycles as described in the Methods. (**a**) Secreted IL-8 protein was detected in the culture medium using the enzyme-linked immunosorbent assay. (**b**) RNA was isolated for the analysis of IL-8 gene expression by RT/real-time PCR. Note: The data are presented as the means and the standard errors of three independent experiments, * *p* < 0.05 vs. normoxia; † *p* < 0.05 vs. one IH cycle; ‡ *p* < 0.05 vs. three IH cycles. Abbreviations: IH = intermittent hypoxia; IL = interleukin.

Monocytic THP-1 cells were treated with normoxia or intermittent hypoxia for one, three and six cycles. Results of the ELISA analysis comparing the secreted proteins isolated from the culture medium of THP-1 cells with or without intermittent hypoxia revealed an increase in the IL-8 protein level by intermittent hypoxia (Figure 1a). Besides that, as shown in Figure 1b, intermittent hypoxia also increased the IL-8 mRNA expression in the monocytic THP-1 cells. The increased expression of IL-8 at both the protein and mRNA levels in monocytes was positively well-correlated with the cycle number of intermittent hypoxia.

### 2.2. Increased IL-8 in the Conditioned Medium of the Intermittent Hypoxia-Treated Cells Promoted the Chemotactic Migration of Monocytes

**Figure 2:**
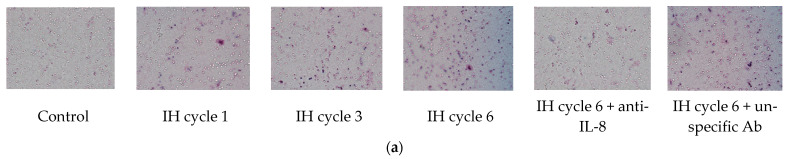

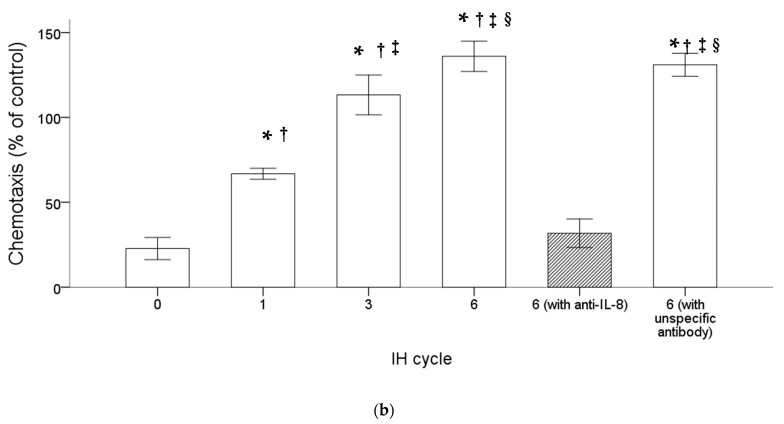
Intermittent hypoxia increased chemotaxis of monocytes with IL-8 dependence. THP-1 cells were treated with normoxia or intermittent hypoxia for one, three and six cycles, and the culture medium was collected as the attractant for the following chemotaxis process. Chemotaxis of the monocytic THP-1 cells toward IL-8 was analyzed by means of the transwell migration assay for 1 h toward the condition medium of the IH-treated THP-1 cells. (**a**,**b**) The number of THP-1 cells that were attracted by IL-8 and migrated through the transwell filter was increased by intermittent hypoxia and diminished by the anti-IL-8 antibody-pretreated conditioned medium. A control experiment to exclude the pure IgG effect with an unspecific antibody “mouse IgG1, Kappa Monoclonal (NCG01)-Isotype Control-BSA and Azide Free” was conducted, which showed no effect on the IL-8-induced migration. Note: The data were presented as the means and the standard errors from three independent experiments, * *p* < 0.05 vs. normoxia; † *p* < 0.05 vs. six IH cycles with anti-IL-8; ‡ *p* < 0.05 vs. one IH cycle; § *p* < 0.05 vs. three IH cycles. Abbreviations: IH = intermittent hypoxia; IL = interleukin.

THP-1 cells were treated with normoxia or intermittent hypoxia for one, three and six cycles, and the culture medium was collected as the attractant for the following chemotactic migration assay. The chemotactic migration of the monocytic THP-1 cells toward the lower chamber which contained the conditioned medium of the intermittent hypoxia-treated cells was analyzed by means of the transwell migration assay for 1 h. The number of the THP-1 cells that migrated through the transwell filter was significantly increased when the lower chamber contained the medium collected from the intermittent hypoxia-treated cells, and this chemotactic effect was positively well-correlated with the number of IH cycles (Figure 2a,b). The IH-induced chemotactic migration described above was diminished when the conditioned medium was pretreated with the anti-IL-8 antibody (Figure 2a,b).

### 2.3. Intermittent Hypoxia Induced IL-8 Production through the ERK, PI3K, PKC and NF-κB Signal Pathways in the Monocytic THP-1 Cells

**Figure 3:**
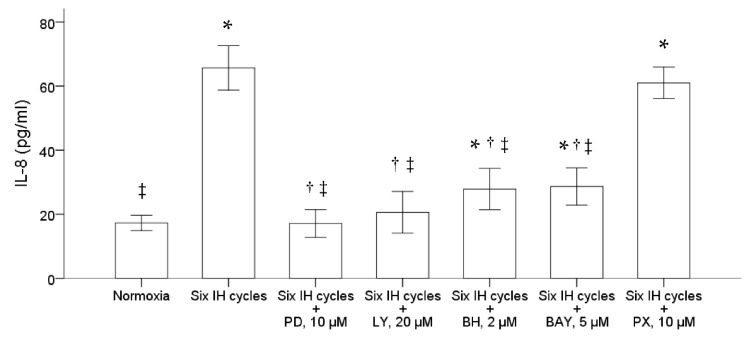
Intermittent hypoxia induced activation of the ERK, PI3K, PKC and NF-κB signal pathways in the THP-1 cells. The THP-1 cells were treated with normoxia or six cycles of intermittent hypoxia and the culture medium was collected for the enzyme-linked immunosorbent assay. PD98059, LY294002, bisindolylmaleimide I hydrochloride, Bay11-7082 and PX-478, inhibitors specific for the ERK, PI3K, PKC, NF-κB and HIF-1α pathways, respectively, were then used to pretreat the monocytic THP-1 cells one hour before the condition of intermittent hypoxia. The results demonstrated that pretreatment with either 10 μM PD98059, 20 μM LY294002, 2 μM bisindolylmaleimide I hydrochloride and 5 μM Bay11-7082 diminished the IL-8 production induced by intermittent hypoxia. Note: The data were presented as the means and the standard errors from three independent experiments, * *p* < 0.05 vs. normoxia; † *p* < 0.05 vs. six IH cycles; ‡ *p* < 0.05 vs. six IH cycles + PX 10 μM. Abbreviations: BAY = Bay11-7082; BH = bisindolylmaleimide I hydrochloride; IH = intermittent hypoxia; IL = interleukin; LY = LY294002; PD = PD98059; PX = PX-478.

We further examined the possible pathway involved in the upregulation of IL-8 production in the monocytic THP-1 cells by intermittent hypoxia. Before the stimulation of intermittent hypoxia, the THP-1 cells were treated for one hour with PD98059, LY294002, bisindolylmaleimide I hydrochloride and Bay11-7082, specific for inhibiting the activation of ERK, PI3K, PKC and NF-κB, respectively. The results demonstrated that pretreatment with either 10 μM PD98059, 20 μM LY294002, 2 μM bisindolylmaleimide I hydrochloride or 5 μM Bay11-7082 significantly suppressed the induction of IL-8 induced by intermittent hypoxia (Figure 3). Pretreatment with PX-478, a specific inhibitor of HIF-1α, did not affect the induction of IL-8 by intermittent hypoxia in the THP-1 cells.

### 2.4. Increase in the Plasma IL-8 Level and Monocytic IL-8 mRNA Expression after One Night’s Sleep in OSA Patients

**Figure 4:**
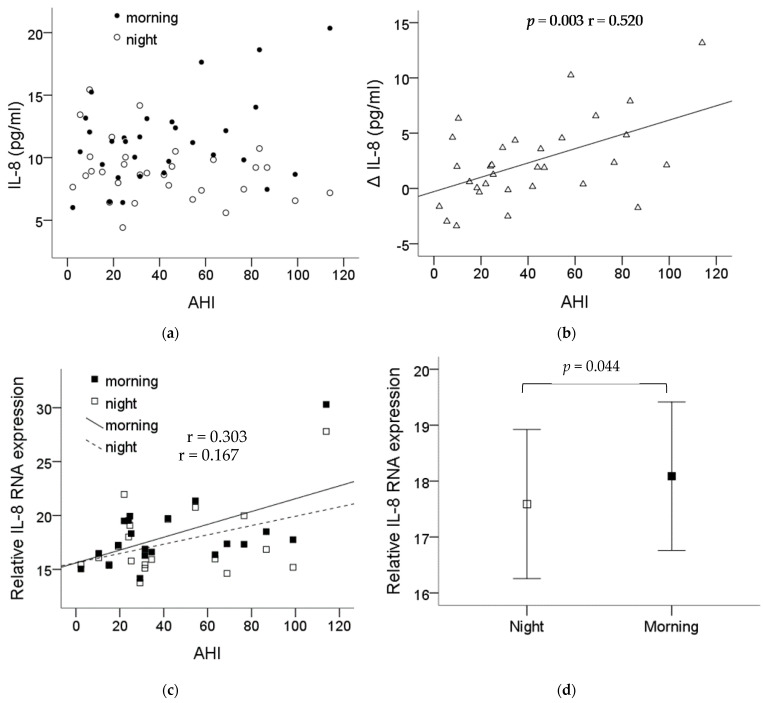
IL-8 expression significantly increased in the plasma and the monocytes of the OSA patients. Plasma was collected before and after the night PSG study, then submitted for monocyte isolation. (**a**) The levels of plasma IL-8 from each patient before and after the PSG study. (**b**) The difference in the plasma IL-8 levels before and after one night’s sleep was presented with ΔIL-8 which showed significant correlation with the severity of OSA (*p* = 0.003, r = 0.520). (**c**) The monocytes IL-8 mRNA expression was also found to be increased along the severity of the OSA patients’ condition. (**d**) The expression of the monocytes’ IL-8 mRNA was elevated with statistical significance comparing the expression before and after one night’s sleep (*p* = 0.044). Note: The data were presented as the means and the standard errors. Abbreviations: AHI = apnea–hypopnea index; IL = interleukin.

The demographic data of the thirty-one recruited patients are shown in Table 1. Blood was collected before and after the night PSG study, then submitted for monocyte isolation. The plasma IL-8 levels in the patients are shown in Figure 4a. The difference (ΔIL-8) in the plasma IL-8 levels before and after one night’s sleep showed a significant correlation with the severity of OSA (*p* = 0.003, r = 0.520) (Figure 4b). The monocytes’ IL-8 mRNA expression was also found to be increased along the severity of the OSA patients’ condition (Figure 4c) with statistical significance comparing the expression before and after one night’s sleep, *p* = 0.044 (Figure 4d).

## 3. Discussion

In this study, we demonstrated that intermittent hypoxia can upregulate the expression of IL-8 in monocytic THP-1 cells at both the secreted protein and mRNA levels, which subsequently increases the chemotactic migration of monocytic THP-1 cells. Besides, the ERK, PI3K, PKC and NF-κB pathways were revealed to contribute to the activation of monocytic THP-1 cells induced by intermittent hypoxia. Furthermore, monocytic IL-8 expression at both the protein and mRNA levels in OSA patients were increased overnight and positively correlated well with disease severity.

IL-8 has been considered to be a chemoattractant for neutrophils, which are cells that actively participate in the first line of defense in the immune system [22]. Nevertheless, IL-8 has also been documented to be responsible for the attraction, adhesion and migration of monocytes and mediate the accumulation of macrophages in atherosclerotic lesions [15,23]. By cooperation with other chemokines, IL-8 was reported to be involved in the initial contact of monocytes with the endothelium and MCP-1 participated in transmigration, whereas both MCP-1 and IL-8 are involved in the adhesion of monocytes to the endothelium [16,17]. Although some literature sources have demonstrated the increased circulating IL-8 levels in OSA patients [17,18,19], one study published by Kim et al. showed that the serum concentrations of IL-8 did not differ between the OSA patients and the normal controls while the plasma MCP-1 and adiponectin levels differed between the OSA patients and the normal controls [24]. In our study, we demonstrated the increase in the plasma IL-8 levels and mRNA expression in monocytes of OSA patients. Together with our previous finding that MCP-1 expression is significantly higher in OSA patients [25], the increase in IL-8 could synergistically enhances the chemotactic migration and adhesion of monocytes to vascular endothelial cells.

Although the increased circulating IL-8 levels in OSA patients were documented in some literature sources [19,20,21], the possible pathophysiologic mechanism related to intermittent hypoxia has not been proven yet [26,27]. One study demonstrated the levels of proinflammatory cytokines IL-8 secreted from human aortic endothelial cells was elevated under the intermittent hypoxia condition [28]. Another study showed the increase in inflammatory signals including IL-8 in lymphocytes from intermittent hypoxia-exposed rats [29]. A recent study revealed enhanced IL-8 production in mononuclear cells in young children with severe obstructive sleep apnea which was speculated to be associated with sleep-related chronic intermittent hypoxia [30]. Our study is the first one to demonstrate that in vitro intermittent hypoxia can promote the IL-8 expression in monocytes at both the secreted protein and mRNA levels. The pro-atherosclerotic effect of intermittent hypoxia was also investigated in terms of modulating the IL-8-induced chemotactic migration of monocytes. In addition, we further examined the changes of monocytic IL-8 expression in OSA patients and found that apnea events occurring during overnight sleep can enhance the expression of IL-8 in monocytes.

The activation of IL-8 gene expression in monocytes has been reported to be dependent on the regulation of signal pathways including ERK and PI3K [31]. Pretreatment with PD98059 and LY294002 to inhibit ERK and PI3K in monocytes decreased the IL-8 gene expression induced by different stimulators [31,32]. The IL-8 production was also proved to be regulated by PKC in human keratinocytes, synovial fibroblasts and breast cancer cells [33,34,35]. However, the regulatory pathway of monocytic IL-8 gene expression under intermittent hypoxia conditions has not been investigated. In our study, pretreatment with PD98059, LY294002 and bisindolylmaleimide I hydrochloride which inhibit the ERK, PI3K and PKC pathways, respectively, suppressed the monocytic IL-8 expression induced by intermittent hypoxia. The results demonstrated the activation of ERK, PI3K and PKC was required for the increased IL-8 expression in monocytes induced by intermittent hypoxia.

Our study also showed that Bay11-7082, an NF-κB inhibitor, can significantly reduce the IH-induced IL-8 elevation, which means the activation of NF-κB is required for the IH-induced IL-8 upregulation. Together with the activation of ERK, it suggests that oxidative stress in monocytes is likely involved in this mechanism [36]. The syndrome of obstructive sleep apnea during hypopnea/apnea events, poor alveolar ventilation reduce the oxygen saturation in arterial blood and lead to a consequent oxidative imbalance as a result of intermittent hypoxia, with production of reactive oxygen species, the factors of tumor necrosis, inflammatory cytokines (IL-2, IL-4, IL-6), lipid peroxidation and cell-free DNA. Such molecules could act as severity biomarkers [37]. An oxidative/reductive imbalance was noted in the uvula mucosa of individuals with OSA and the total antioxidant status of the uvula mucosa suppressed in the uvular mucosa is associated with the onset of OSA [38].

Hypoxia indicator HIF-1α is known to play an important role in hypoxia response [39]. However, the pretreatment of the THP-1 cells with PX-478, a specific HIF-1α inhibitor, did not interfere with the increase in IL-8 under IH condition. A previous study demonstrated selective activation of inflammatory overadaptive pathways in the intermittent hypoxia condition and OSA [40]. They found in vitro intermittent hypoxia selectively activates NF-κB-dependent transcription, but not that hypoxia activates HIF-1α-dependent transcription. This finding might explain the results of a recently published study of OSA patients. Serum HIF-1α protein levels remained chronically upregulated through sustained hypoxia, but had no difference between the evening and the morning values through intermittent hypoxia [41]. The same reason can be applied to the fact that one-night CPAP treatment cannot decrease the elevated serum HIF-1-alpha levels but long-term, e.g., 2-month-long, CPAP treatment can significantly decrease the elevated serum HIF-1-alpha levels [42,43].

The severity-dependent increased IL-8 gene expression in monocytes of OSA patients was confirmed in this study. More importantly, we spent effort on collecting and purifying the monocytes immediately from fresh blood before and after PSG which lead us to find out that one night’s sleep with intermittent hypoxia can result in the upregulation of IL-8 production in monocytes. We also established a well-controlled chamber for culturing cells under the intermittent hypoxia condition and the IH setting could successfully induce the IL-8 gene expression in monocytes which nicely mimics the in vivo condition. By using this in vitro cell model, one can directly study the potential effect of intermittent hypoxia on cells and the underlying mechanism. However, some limitations still need to be mentioned in this study. The relatively small OSA case numbers in our study were due to the stringent inclusion criteria. The possible confounders, such as ischemic heart disease and other inflammatory disease, that could influence the IL-8 expression were excluded during enrollment. In addition, the lack of the normal controls comparing to the OSA patients was due to the fact that individuals with relatively high BMI tend to have some respiratory events during sleep. As we know, sleep-disordered breathing is a spectrum of disorders, from simple snoring and upper airway resistance to obstructive sleep apnea [44]. Even in the mildest form of simple snoring, intermittent hypoxia did happen and lead to subsequent sequelae [45]. Our study, although without perfect normal controls, still confirmed the positive linear regression between the elevated plasma IL-8 levels, monocytic IL-8 expression and severity of OSA.

In conclusion, this study for the first time demonstrates that intermittent hypoxia can enhance the IL-8 gene expression, protein secretion and the subsequent chemotactic migration ability of monocytes. The ERK, PI3K, PKC and NF-κB signaling pathways are documented to be involved in the IL-8 expression of monocytes upregulated by intermittent hypoxia. Furthermore, monocytic IL-8 expression in the OSA patients was found to be elevated after one night’s sleep and positively dependent on disease severity. These findings point out the important role of IL-8 which is responsible for the increased chemotactic migration of monocytes under the intermittent hypoxia condition. It is possible that blocking the IL-8 function with antagonists to reduce the intermittent hypoxia-induced chemotactic migration of monocytes, an early inflammatory process of atherosclerosis, could be one potential strategy to reduce the progression of atherosclerosis in patients with OSA.

## 4. Materials and Methods

### 4.1. Materials

The monoclonal antibody against IL-8 was obtained from Epitomics Inc. (Burlingame, CA, USA). Recombinant IL-8 was purchased from R&D Systems Inc. (Minneapolis, MO, USA). PI3K inhibitor LY294002 and ERK inhibitor PD98059 were purchased from SIGMA Inc. (Marlborough, MO, USA). PKC inhibitor bisindolylmaleimide I hydrochloride was purchased from SIGMA Inc. (Marlborough, MO, USA). NF-κB inhibitor Bay11-7082 was purchased from SIGMA Inc. (Marlborough, MO, USA), and HIF-1 inhibitor PX-478 was purchased from Cayman Chemical Co. (Jamestown, MI, USA). The unspecific antibody for the control experiment, mouse IgG1, Kappa Monoclonal (NCG01)-Isotype Control-BSA and Azide Free, was purchased from Abcam PLC. (Cambridge, UK).

### 4.2. Monocyte Culture

THP-1, the human monocytic leukemia cell line, was obtained from ATCC (Taiwan). The THP-1 cells were grown in the suspension culture of the RPMI 1640 medium supplemented with antibiotics and 10% fetal bovine serum. The cells were grown at 37 °C in a humidified atmosphere with 5% CO_2_/95% air and subcultured by diluting the medium with a fresh growth medium in a 1:4 ratio.

### 4.3. Intermittent Hypoxia Culture Conditions

Monocytic THP-1 cells (1 × 10^6^ cells/mL) were resuspended in a 5 cm culture dish containing 5 mL RPMI 1640 medium. The condition of intermittent hypoxia was performed in a customized gas flow chamber modified from the Hypo-Hyper Oxygen System, NexBioxy Inc. (Hsinchu County, Taiwan). As described previously [46], the monocytic THP-1 cells were placed in either condition of normoxia (21% O_2_ with 5% CO_2_ and balance with N_2_) or intermittent hypoxia (35 min of hypoxia (0.1% O_2_ with 5% CO_2_ and balance with N_2_) followed by 25 min of returned normoxia (21% O_2_ with 5% CO_2_ and balance with N_2_) for one cycle) for up to six cycles. The cells in the intermittent hypoxia chamber were then maintained in a standard incubator (21% O_2_ with 5% CO_2_ and balance with N_2_), Thermo Inc. (Waltham, OH, USA), at 37 °C for another 18 h before the following analysis.

### 4.4. RNA Extraction and RT/Real-Time PCR

As described previously [25], total cellular RNA was isolated by means of lysis in a guanidinium isothiocyanate buffer, followed by a single step of phenol–chloroform–isoamyl alcohol extraction. The cDNA was synthesized from total RNA using the M-MLV reverse transcriptase, USB Corporation (Cleveland, OH, USA). The PCR primers used were as follows: GAPDH forward primer 5′-GACCTGACCTGCCGTCTA-3′ and reverse primer 5′-AGGAGTGGGTGTCGCTGT-3′ and IL-8 forward primer 5′-GCTGTGTTTGCGTCTCTCCCAGGA-3′ and IL-8 reverse primer 5′-CTCACAGCCCTGTGCCTCTTCTTC-3′. Quantitative real-time PCR was performed with the universal cycling conditions (15 min at 95 °C, followed by 40 cycles of 30 s at 95 °C, 1 min at 55 °C and 30 s at 72 °C). The cycle threshold (CT) values were determined by means of automated threshold analysis with the Mx-Pro Mx3005P v4.00 software, Agilent Tech. (Santa Clara, CA, USA).

### 4.5. Enzyme-Linked Immunosorbent Assay (ELISA)

The levels of IL-8 in the plasma and the culture medium were determined using the ELISA kits obtained from R&D System, Inc. (Minneapolis, MO, USA). A 96-well microplate was coated with the diluted capture antibody overnight at room temperature. After washing, the microplate was blocked with 300 μL of a reagent diluent for 1 h. The samples and the standards, after dilution with the diluent reagent, were added to the microplate and incubated for 2 h. After washing, 100 μL of the detection antibody was added and incubated for 2 h. Thereafter, 100 μL of streptavidin–HRP (horseradish peroxidase) was added and incubated for 20 min. Then, 100 μL of the substrate solution were added and incubated for 20 min. The final reaction was stopped by adding 50 μL stop solution. Using a microplate reader, the concentration of IL-8 was determined by absorbance at 450 nm.

### 4.6. Cell Migration Assay

Microporous membrane transwell inserts (8 μm pore size, Costar, Washington, DC, USA) were used for the chemotaxis assay; 2 × 10^5^ THP-1 cells in 200 μL RPMI were added to the upper chamber and the lower chamber containing the conditioned medium obtained from the THP-1 cell cultures after different cycles of intermittent hypoxia as attractants. The cells were allowed to migrate for 1 h and the nonmigratory cells were removed before the membrane was mounted. The number of migratory cells was revealed by Liu’s stain and counted under a microscope as described previously [25].

### 4.7. Clinical Patients

**Figure 5:**
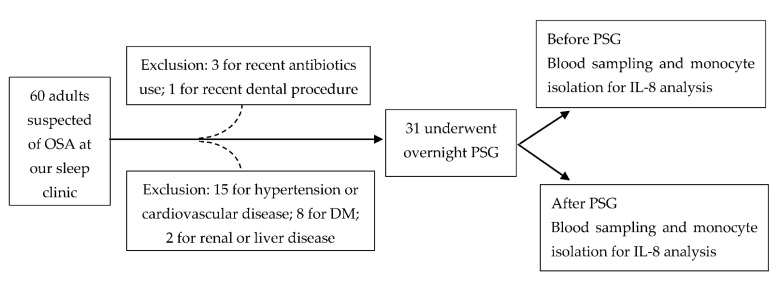
The flow chart of our patients’ selection and study protocol. Abbreviations: DM = diabetes mellitus; IL = interleukin; OSA: obstructive sleep apnea; PSG: polysomnography.

In this study, 60 adult (>20 years old) patients, under the suspicion of possible OSA, were included from our sleep center initially. The sample size was calculated based on the changes of IL-8 in OSA patients from the published literature [20]. We used G*power (Version 3.1.9.7 for Windows) with the setting for power = 0.8, two-tailed alpha error = 0.05 and the calculated effect size = 0.518 (mean H0 = 3.124, mean H1 = 5.308 and SD = 4.216 from [20]) and obtained the total sample size of 32 subjects. We estimated the sample size missing rate at 40%, therefore, we needed at least 54 subjects enrolled in this study. The exclusion criteria included recent (<1 month) or chronic significant infectious or inflammatory condition, including trauma, invasive medical/surgical/dental procedure; coexistence of ischemic heart disease, hypertension, diabetes, hyperlipidemia, cerebrovascular disease, liver disease or renal disease; recent use (<1 month) of antibiotics or anti-inflammatory drugs. Finally, 31 patients underwent the study protocols. The flow chart of our patients’ selection and study protocol is shown in Figure 5. The Institutional Review Board of Chang Gung Memorial Hospital (Nos. 104-9739B and 201601727A3) approved this study, and written informed consent was obtained from every participant before the study.

### 4.8. Polysomnography

All the patients were examined by means of standard in-laboratory overnight polysomnography (PSG) with Embla N7000 (Medcare, Reykjavik, Iceland). The variables recorded were four channels of the electroencephalogram (C3/A2, C4/A1, O1/A2, O2/A1); bilateral electrooculogram; chin, left and right anterior tibial electromyogram; electrocardiogram; airflow (measured by flow sensors and thermistors); chest and abdominal wall movement (measured by inductive plethysmographic bands); snoring (measured using a neck microphone); and arterial oxygen saturation (SpO_2_) (measured by finger pulse oximetry). All the measurements were collected in a computerized sleep system with Somnologica Studio 3.0 (Medcare, Reykjavik, Iceland). Apnea was defined as cessation of airflow for at least 10 s, and hypopnea was defined as an abnormal respiratory event with at least a 30% reduction in airflow (relative to the baseline) for at least 10 s, with at least 3% oxygen desaturation and/or arousal. The apnea–hypopnea index (AHI) is the number of events of apnea plus hypopnea per hour of total sleep time. The oxygen desaturation index (ODI) was the number of times per hour during sleep that the blood oxygen level dropped by 3% or more from the baseline [47].

### 4.9. Blood Sampling and Monocyte Isolation

Peripheral venous blood (20 mL) was sampled at 10 pm, just before the PSG study, and at 6 am the next morning when the patients woke up after PSG was finished, in supine position under the fasting condition. Tubes rinsed with heparin were used to collect peripheral blood samples and centrifugation at 3000× *g* rpm was immediately carried out for 20 min and the plasma was used for the analysis of secreted IL-8. Mononuclear cells in the blood cells were then isolated by means of Ficoll–Hypaque centrifugation and CD14+ monocytes were enriched by using an autoMACS magnetic cell sorting system (Miltenyi Biotec, Bergisch Gladbach, Germany) as described previously [48].

### 4.10. Statistical Analysis

A *t*-test was used to compare the mean value of the IL-8 protein or gene expression between the two groups, such as different cycles of intermittent hypoxia or diurnal changes. Linear regression was used to test the relationship between the IL-8 protein or gene expression and the AHI, to see the changes in IL-8 expression with the severity of OSA. All the statistical tests were performed with the use of the SPSS software (SPSS Institute, Chicago, IL, USA). A *p*-value of 0.05 or less was considered to indicate statistical significance, and all the data were expressed as the means ± SEM.

## Figures and Tables

**Table 1:** Demographic data and polysomnography parameters of the enrolled OSA patients.

Number of subjects (male)	31 (25)
Age, years	44.3 ± 4.8
BMI, kg/m^2^	26.9 ± 2.5
AHI, events/hour	42.8 ± 10.5
Sleep efficiency, %	71.2 ± 5.9
ODI, events/hour	39.6 ± 6.7
Mean SpO_2_, %	88.4 ± 5.2
Lowest SpO_2_, %	75.1 ± 8.8
Time with SpO_2_ < 85%, minutes	12.9 ± 9.8

Note: The data were presented as the means and the standard errors. Abbreviations: BMI = body mass index; AHI = apnea–hypopnea index; ODI = 3% oxygen desaturation index; SpO_2_ = oxygen saturation.

## Data Availability

Data sharing is not applicable to this article.

## References

[1] Peppard P.E., Young T., Barnet J.H., Palta M., Hagen E.W., Hla K.M. (2013). Increased prevalence of sleep-disordered breathing in adults. Am. J. Epidemiol..

[2] Jordan A.S., McSharry D.G., Malhotra A. (2014). Adult obstructive sleep apnoea. Lancet.

[3] Peters R.W. (2005). Obstructive sleep apnea and cardiovascular disease. Chest.

[4] Suri T.M., Suri J.C. (2021). A review of therapies for the overlap syndrome of obstructive sleep apnea and chronic obstructive pulmonary disease. FASEB Bioadv..

[5] Brodie K.D., Goldberg A.N. (2021). Obstructive Sleep Apnea: A Surgeon’s Perspective. Med. Clin. N. Am..

[6] Suslu A.E., Pamuk G., Pamuk A.E., Ozer S., Jafarov S., Onerci T.M. (2017). Effects of Expansion Sphincter Pharyngoplasty on the Apnea-Hypopnea Index and Heart Rate Variability. J. Oral Maxillofac. Surg..

[7] Iannella G., Magliulo G., Di Luca M., De Vito A., Meccariello G., Cammaroto G., Pelucchi S., Bonsembiante A., Maniaci A., Vicini C. (2020). Lateral pharyngoplasty techniques for obstructive sleep apnea syndrome: A comparative experimental stress test of two different techniques. Eur. Arch. Otorhinolaryngol..

[8] Pietila K., Tenkanen L., Manttari M., Manninen V. (1997). How to define coronary heart disease in register-based follow-up studies: Experience from the Helsinki Heart Study. Ann. Med..

[9] Maekawa M., Shiomi T., Usui K., Sasanabe R., Kobayashi T. (1998). Prevalence of ischemic heart disease among patients with sleep apnea syndrome. Psychiatry Clin. Neurosci..

[10] Hung J., Whitford E.G., Parsons R.W., Hillman D.R. (1990). Association of sleep apnoea with myocardial infarction in men. Lancet.

[11] Nacher M., Farre R., Montserrat J.M., Torres M., Navajas D., Bulbena O., Serrano-Mollar A. (2009). Biological consequences of oxygen desaturation and respiratory effort in an acute animal model of obstructive sleep apnea (OSA). Sleep Med..

[12] Mestas J., Ley K. (2008). Monocyte-endothelial cell interactions in the development of atherosclerosis. Trends Cardiovasc. Med..

[13] Hansson G.K. (2009). Inflammatory mechanisms in atherosclerosis. J. Thromb. Haemost..

[14] Pease J.E., Sabroe I. (2002). The role of interleukin-8 and its receptors in inflammatory lung disease: Implications for therapy. Am. J. Respir. Med..

[15] Terkeltaub R., Boisvert W.A., Curtiss L.K. (1998). Chemokines and atherosclerosis. Curr. Opin. Lipidol..

[16] Boisvert W.A. (2004). The participation of chemokines in atherosclerosis. Discov. Med..

[17] Gerszten R.E., Garcia-Zepeda E.A., Lim Y.C., Yoshida M., Ding H.A., Gimbrone M.A., Luster A.D., Luscinskas F.W., Rosenzweig A. (1999). MCP-1 and IL-8 trigger firm adhesion of monocytes to vascular endothelium under flow conditions. Nature.

[18] Boisvert W.A., Santiago R., Curtiss L.K., Terkeltaub R.A. (1998). A leukocyte homologue of the IL-8 receptor CXCR-2 mediates the accumulation of macrophages in atherosclerotic lesions of LDL receptor-deficient mice. J. Clin. Investig..

[19] Ohga E., Tomita T., Wada H., Yamamoto H., Nagase T., Ouchi Y. (2003). Effects of obstructive sleep apnea on circulating ICAM-1, IL-8, and MCP-1. J. Appl. Physiol..

[20] Alzoghaibi M.A., Bahammam A.S. (2005). Lipid peroxides, superoxide dismutase and circulating IL-8 and GCP-2 in patients with severe obstructive sleep apnea: A pilot study. Sleep Breath.

[21] Carpagnano G.E., Spanevello A., Sabato R., Depalo A., Palladino G.P., Bergantino L., Foschino Barbaro M.P. (2010). Systemic and airway inflammation in sleep apnea and obesity: The role of ICAM-1 and IL-8. Transl. Res..

[22] Soehnlein O., Lindbom L., Weber C. (2009). Mechanisms underlying neutrophil-mediated monocyte recruitment. Blood.

[23] Murphy N., Bruckdorfer K.R., Grimsditch D.C., Overend P., Vidgeon-Hart M., Groot P.H., Benson G.M., Graham A. (2003). Temporal relationships between circulating levels of CC and CXC chemokines and developing atherosclerosis in apolipoprotein E*3 Leiden mice. Arterioscler. Thromb. Vasc. Biol..

[24] Kim J., Lee C.H., Park C.S., Kim B.G., Kim S.W., Cho J.H. (2010). Plasma levels of MCP-1 and adiponectin in obstructive sleep apnea syndrome. Arch. Otolaryngol. Head Neck Surg.

[25] Chuang L.P., Chen N.H., Lin Y., Ko W.S., Pang J.H. (2016). Increased MCP-1 gene expression in monocytes of severe OSA patients and under intermittent hypoxia. Sleep Breath.

[26] Tamisier R., Pepin J.L., Remy J., Baguet J.P., Taylor J.A., Weiss J.W., Levy P. (2011). 14 nights of intermittent hypoxia elevate daytime blood pressure and sympathetic activity in healthy humans. Eur. Respir. J..

[27] Querido J.S., Sheel A.W., Cheema R., Van Eeden S., Mulgrew A.T., Ayas N.T. (2012). Effects of 10 days of modest intermittent hypoxia on circulating measures of inflammation in healthy humans. Sleep Breath.

[28] Polotsky V.Y., Savransky V., Bevans-Fonti S., Reinke C., Li J., Grigoryev D.N., Shimoda L.A. (2010). Intermittent and sustained hypoxia induce a similar gene expression profile in human aortic endothelial cells. Physiol. Genom..

[29] Guo H., Cao J., Li J., Yang X., Jiang J., Feng J., Li S., Zhang J., Chen B. (2015). Lymphocytes from intermittent hypoxia-exposed rats increase the apoptotic signals in endothelial cells via oxidative and inflammatory injury in vitro. Sleep Breath.

[30] Ke D., Kitamura Y., Lejtenyi D., Mazer B., Brouillette R.T., Brown K. (2019). Enhanced interleukin-8 production in mononuclear cells in severe pediatric obstructive sleep apnea. Allergy Asthma Clin. Immunol..

[31] Syeda F., Liu H.Y., Tullis E., Liu M., Slutsky A.S., Zhang H. (2008). Differential signaling mechanisms of HNP-induced IL-8 production in human lung epithelial cells and monocytes. J. Cell Physiol..

[32] Selvaraj S.K., Giri R.K., Perelman N., Johnson C., Malik P., Kalra V.K. (2003). Mechanism of monocyte activation and expression of proinflammatory cytochemokines by placenta growth factor. Blood.

[33] Chabot-Fletcher M., Breton J., Lee J., Young P., Griswold D.E. (1994). Interleukin-8 production is regulated by protein kinase C in human keratinocytes. J. Investig. Dermatol..

[34] Jordan N.J., Watson M.L., Yoshimura T., Westwick J. (1996). Differential effects of protein kinase C inhibitors on chemokine production in human synovial fibroblasts. Br. J. Pharmacol..

[35] Chou W.Y., Chuang K.H., Sun D., Lee Y.H., Kao P.H., Lin Y.Y., Wang H.W., Wu Y.L. (2015). Inhibition of PKC-Induced COX-2 and IL-8 Expression in Human Breast Cancer Cells by Glucosamine. J. Cell Physiol..

[36] Chai W., Zhang J., Duan Y., Pan D., Liu W., Li Y., Yan X., Chen B. (2014). Pseudomonas pyocyanin stimulates IL-8 expression through MAPK and NF-kappaB pathways in differentiated U937 cells. BMC Microbiol..

[37] Maniaci A., Iannella G., Cocuzza S., Vicini C., Magliulo G., Ferlito S., Cammaroto G., Meccariello G., De Vito A., Nicolai A. (2021). Oxidative Stress and Inflammation Biomarker Expression in Obstructive Sleep Apnea Patients. J. Clin. Med..

[38] Olszewska E., Rogalska J., Brzoska M.M. (2021). The Association of Oxidative Stress in the Uvular Mucosa with Obstructive Sleep Apnea Syndrome: A Clinical Study. J. Clin. Med..

[39] Hu C.J., Wang L.Y., Chodosh L.A., Keith B., Simon M.C. (2003). Differential roles of hypoxia-inducible factor 1alpha (HIF-1alpha) and HIF-2alpha in hypoxic gene regulation. Mol. Cell Biol..

[40] Ryan S., Taylor C.T., McNicholas W.T. (2005). Selective activation of inflammatory pathways by intermittent hypoxia in obstructive sleep apnea syndrome. Circulation.

[41] Gabryelska A., Szmyd B., Szemraj J., Stawski R., Sochal M., Bialasiewicz P. (2020). Patients with obstructive sleep apnea present with chronic upregulation of serum HIF-1alpha protein. J. Clin. Sleep Med..

[42] Lu D., Li N., Yao X., Zhou L. (2017). Potential inflammatory markers in obstructive sleep apnea-hypopnea syndrome. Bosn. J. Basic Med. Sci..

[43] Gabryelska A., Stawski R., Sochal M., Szmyd B., Bialasiewicz P. (2020). Influence of one-night CPAP therapy on the changes of HIF-1alpha protein in OSA patients: A pilot study. J. Sleep Res..

[44] Stoohs R.A., Knaack L., Blum H.C., Janicki J., Hohenhorst W. (2008). Differences in clinical features of upper airway resistance syndrome, primary snoring, and obstructive sleep apnea/hypopnea syndrome. Sleep Med..

[45] Urschitz M.S., Guenther A., Eggebrecht E., Wolff J., Urschitz-Duprat P.M., Schlaud M., Poets C.F. (2003). Snoring, intermittent hypoxia and academic performance in primary school children. Am. J. Respir. Crit. Care Med..

[46] Chuang L.P., Chen N.H., Lin S.W., Hu H.C., Kao K.C., Li L.F., Yang C.T., Huang C.C., Pang J.S. (2019). Monocytic C-C chemokine receptor 5 expression increases in in vitro intermittent hypoxia condition and in severe obstructive sleep apnea patients. Sleep Breath.

[47] Berry R.B., Budhiraja R., Gottlieb D.J., Gozal D., Iber C., Kapur V.K., Marcus C.L., Mehra R., Parthasarathy S., Quan S.F. (2012). Rules for scoring respiratory events in sleep: Update of the 2007 AASM Manual for the Scoring of Sleep and Associated Events. Deliberations of the Sleep Apnea Definitions Task Force of the American Academy of Sleep Medicine. J. Clin. Sleep Med..

[48] Chuang L.P., Chen N.H., Lin S.W., Chang Y.L., Liao H.R., Lin Y.S., Chao I.J., Lin Y., Pang J.H. (2014). Increased C-C chemokine receptor 2 gene expression in monocytes of severe obstructive sleep apnea patients and under intermittent hypoxia. PLoS ONE.

